# Integrating design thinking and implementation science principles in delivering a medication review service in the community pharmacy setting—An implementation testing study

**DOI:** 10.1371/journal.pone.0304291

**Published:** 2024-06-13

**Authors:** Maali Mustafa, Ernieda Hatah, Mohd Makmor-Bakry

**Affiliations:** 1 Faculty of Pharmacy, Universiti Kebangsaan Malaysia, Jalan Raja Muda Abdul Aziz, Kuala Lumpur, Malaysia; 2 Faculty of Pharmacy, University of Cyberjaya, Persiaran Bestari, Cyberjaya, Selangor, Malaysia; University of Science and Technology of Fujairah, YEMEN

## Abstract

**Background:**

Medication review (MR) services are evidenced-based practices in which a systematic assessment of a patient’s medication is conducted, primarily aiming to optimize drug therapy and minimize adverse drug events through pharmacist interventions. Although studies show that MR services are effective, the implementation of MR services in Malaysia has been challenging due to several barriers. An MR services blueprint was developed to be adapted to the Malaysian community pharmacy setting as part of tailoring strategies.

**Objective:**

Through utilizing the design thinking triple diamond model and implementation science principles, a powerful guide for healthcare researchers and stakeholders to assist with effective service implementation, this study aimed to evaluate the implementation testing and observe the effectiveness of the developed MR service blueprint.

**Method:**

The study utilizes an effectiveness-implementation Type 3 hybrid implementation science framework conducted from May 2021 to April 2022. Employing a qualitative ethnographic approach, researchers observed pharmacy study sites during the implementation of MR services. Both qualitative and quantitative data were collected across exploration, preparation, testing, and operational phases. Implementation outcomes evaluated include phases, reach, fidelity, acceptability, as well as implementation barriers and strategies. MR intervention outcomes included service characteristics and the number and type of drug-related problems and interventions offered.

**Results:**

17 community pharmacists were invited to pilot the MR service blueprint for six months in their setting. Of this, 78.5% (n = 11) of the pharmacies reached the testing phase, and 36% (n = 4) reached the implementation phase. Fifty-four patients were in the study, giving an implementation reach of 70%. The majority of surveyed patients expressed satisfaction with the service. The total DRP identified was 133, and 64 interventions were provided by the pharmacists. Facilitation strategies such as “Engage stakeholders by creating ownership of the change” and “Equip stakeholders with training” are needed to overcome the barriers.

**Conclusion:**

This study marked the beginning of successful MR service implementation at Malaysian community pharmacies. Future studies with multi-level partnered strategies are required to reach full implementation and sustainability.

## Introduction

The Medication review (MR) service or intervention has evolved as part of the pharmacist-led cognitive services within the shift from product-oriented to patient-oriented care of the pharmacy profession. MR is the most common broad term for this intervention, and its availability and characteristics may differ between countries and healthcare systems [1–3]. The Pharmaceutical Care Network Europe (PCNE) defines MR as “*a structured evaluation of a patient’s medicines with the aim of optimizing medicines use and improving health outcomes*. *This entails detecting drug-related problems and recommending interventions*”[4]. MR is especially useful for patients with chronic diseases who may take various medications and those at a higher risk of medication-related issues [1]. Medication Therapy Management in the United States, Medicine Use Reviews in the United Kingdom and New Zealand, and MedsCheck in Canada and Australia are among the countries that adopt and remunerate systematic MR programs in outpatient and community settings [2, 5–7]. In Malaysia, the Medication Therapy Adherence Clinic (MTAC) has been established since 2004 and is currently practiced in most government hospitals [8]. However, in Malaysia, the MTAC is not compensated nor practiced in the community setting at large [9].

Community pharmacists in Malaysia have been increasingly reported to offer MR services [10]. However, there is currently no standardized model of practices reported to be in use. The MR protocols available in public hospitals and clinics are considered unsuitable for use in community pharmacy settings due to differences in the patient or customer demographics attending these facilities, attributed to the dual healthcare system and lack of dispensing separation, in which a physician who provides a medical prescription can prescribe and dispense medications directly to patients without the patient having to visit a pharmacy. As a result, patients’ ability to obtain MR services is reduced [10–14]. Various studies on MR reported positive clinical, humanistic, and economic outcomes [15–17]. However, it is essential to note that these outcomes observed in trials may not always accurately reflect the real-world practices where such positive results are consistently achieved. In this context, the role of dissemination and implementation science (D&I) becomes crucial as it provides valuable guidance for healthcare researchers and stakeholders to facilitate effective service implementation. Dissemination and implementation science focus on exploring strategies to bridge the gap between research and practice (implementation science) and to disseminate knowledge and information effectively to the practice setting (dissemination science) [18, 19]. By employing these principles, researchers and stakeholders can enhance the successful integration of research findings into practical healthcare applications. The core concepts of service implementation are (1) a process to implement, (2) an innovation (professional pharmacy service), which is influenced across (3) contextual domains by (4) factors, (5) strategies, and (6) evaluations [20, 21]. Using theories, models, and frameworks (TMF), implementation science (IS) often aims to describe, guide, understand, and evaluate implementation [18]. IS TMFs commonly used in healthcare services development include the Consolidated Framework for Implementation Research (CFIR), Promoting Action on Research Implementation in Health Services (PARIHS), Active Implementation Frameworks (AIF), Exploration, Preparation, Implementation, Sustainment (EPIS) framework, and others [22–24]. Moullin et al. articulated meta-frameworks to develop holistic implementation models that guide the implementation and evaluation of pharmacy services in the pharmacy field. These frameworks are known as the "Framework for the Implementation of Services in Pharmacy" (FISpH) and "The Model for the Evaluation of Implementation Programs and Professional Pharmacy Services" [21, 25]. An essential component of IS is implementation strategies, which are defined as "methods or techniques used to enhance the adoption, implementation, and sustainability of a clinical program or practice" [26]. Implementation strategies that consider a change in organizational and behavioral aspects require external facilitators to assist through determining barriers and tailoring strategies and are known as practice change facilitators [27, 28]. The “Expert Recommendations for Implementing Change” (ERIC) are commonly used in IS research and can be applied and tailored depending on the perceived barrier [29, 30]. In the pharmacy field, Moussa et al. reported IS for professional services in the community pharmacy setting, wherein facilitation strategies were categorized to address specific barriers [31]. Regarding evaluation, service effectiveness outcomes are typically assessed in terms of clinical, humanistic, or economic factors. On the other hand, implementation outcomes are measured through various indicators such as fidelity, reach, and acceptability, among others [32]. To comprehensively evaluate service effectiveness and implementation outcomes, an effectiveness-implementation hybrid methodology is often combined [26]. There are three types of hybrid effectiveness-implementation studies based on their primary focus: 1) Hybrid Type 1 primarily evaluates service effectiveness while also observing the implementation process, 2) Hybrid Type 2 evaluates both the effectiveness of the service and the implementation process concurrently, 3) Hybrid Type 3 focuses more on evaluating the implementation process while also observing the effectiveness of the service being implemented [33].

Apart from IS, design thinking is a widely used user-centered approach to address innovation challenges. While IS includes multiple core components and uses evidence-based strategies for successful implementation, design thinking involves understanding the needs and desires of end-users or customers through empathizing, defining, ideating, prototyping, and testing [34]. A well-known design thinking model is the Double Diamond Method. An extension to the model, making it more comprehensive and holistic, has been proposed recently and is known as the Triple Diamond model [35, 36]. The Triple Diamond Model is a comprehensive framework for design and innovation, consisting of three main phases: Discover, Develop, and Deliver. In the Discover phase, research and data collection help understand the problem and set the project’s foundation. This leads to the Develop phase, where ideas are refined into a tangible blueprint. Finally, the Deliver phase rigorously tests this blueprint, ensuring the solution is practical and effective. This model stresses a cyclical process, where insights from later stages refine earlier ones, ensuring a user-centered approach to innovation [35, 36].

Since the development of implementation research and frameworks, the effectiveness-implementation hybrid studies published in the pharmacy field, specifically the community setting, have been conducted in Belgium, Spain, and the USA [37–39]. In Malaysia, multiple trials have provided evidence of the effectiveness of medication review in the community setting [40–42]. The feasibility of brown bag MR in a community pharmacy setting has been explored and reported to be feasible with positive patient feedback. However, besides the small patient sample size (n = 26), implementation process outcome parameters were not evaluated [41]. In Malaysia, MR in the community setting has not been systematically implemented. Using the same protocol as in public health care settings (such as government hospitals and clinics) is challenging due to barriers and setting differences. To initiate change, design thinking and implementation science concepts were integrated and utilized. As part of these concepts, a study was conducted in 2020 to assess MR implementation barriers, facilitators, and potential strategies at the macro level [12]. Based on the feedback received, as a next step in the triple diamond model and a prioritized tailored implementation strategy, the following study defined and developed the MR service blueprint to be adapted to the specific needs of community pharmacy practice in Malaysia. To ensure the success and sustainability of the service outcomes, efficient implementation through implementation testing is crucial. Hence, this study aims to 1- Present the overall meta-framework of the medication review service in Malaysia through the triple diamond design thinking model and implementation science theories, models, and frameworks. 2- Evaluate the implementation process and observe the effectiveness of the developed MR service blueprint.

## Methods

**Fig 1:**
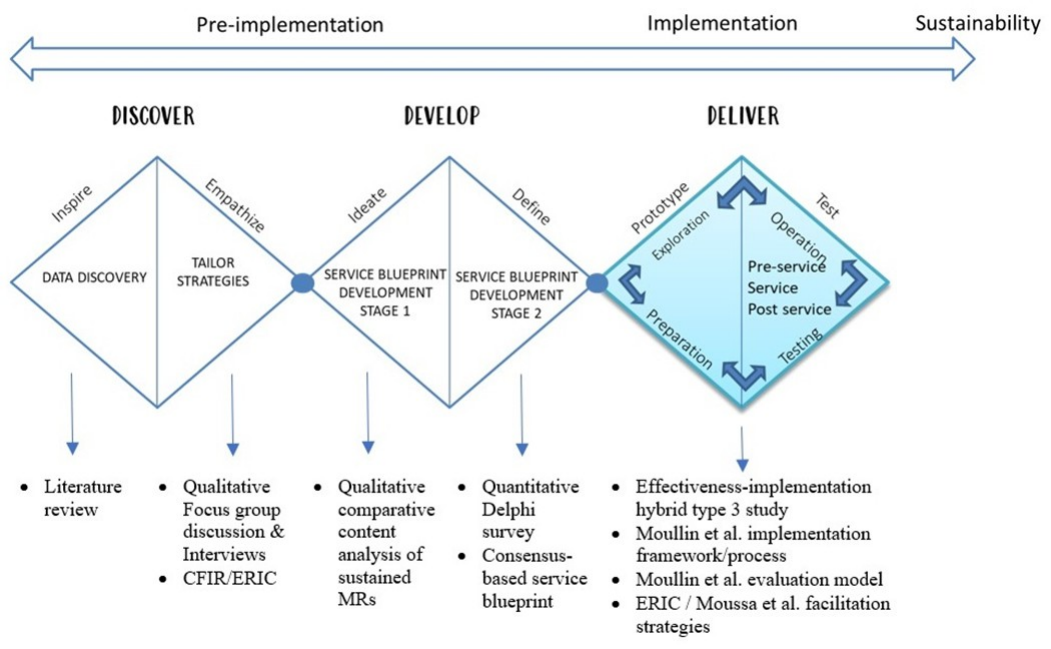
The triple diamond model illustrates the dissemination and implementation of the medication review service in the community setting in Malaysia and the methods and frameworks used in each stage. MR = Medication Review, CFIR = Consolidated Framework for Implementation Research, ERIC = Expert Recommendations for Implementing Change.

This study is part of a bigger project to develop a tailored medication review service implementation model for community pharmacy practice in Malaysia. The triple diamond design thinking model served as a guiding framework, seamlessly integrating all project stages. The model was applied to demonstrate the implementation theories, models, and frameworks utilized throughout the study (Fig 1).

In the discovery stage (first diamond) of the triple diamond method, a literature review followed by a qualitative study was conducted and reported by Mustafa et al. (2022) [12]. The study involved a focus group discussion and individual interviews to assess implementation barriers, facilitators, and strategies. In the development stage (second diamond), a service blueprint was defined and developed using comparative content analysis and an expert consensus (Fig 1). This step is considered as promoting adaptability, which is a prioritized strategy through developing a service blueprint to be adapted to the community setting in Malaysia.

The current paper presents the deliver stage (third diamond), which is the implementation process study phase at the meso/micro level (group and individual pharmacies). The study was designed as a prospective interventional study conducted in community pharmacy settings. It falls under the type 3 hybrid design category, as the primary aim was to evaluate the implementation of the MR intervention while also observing and gathering information on the service’s effectiveness. The implementation study was carried out between May 2021 and April 2022. The study received ethical approval from the Human Research Ethics Committee at the Centre for Research and Instrumentation Management, Universiti Kebangsaan Malaysia (UKM PPI/111/8/JEP-2020-141).

### Implementation study (data collection, sampling, and recruitment)

The study utilized a qualitative ethnographic approach, wherein the researchers observed the community pharmacy study sites as they conducted the medication review service. This approach was chosen to facilitate the evaluation of implementation outcomes, identify barriers, and iteratively tailor strategies to enhance the implementation process. The primary implementation strategy being evaluated is the medication review blueprint developed to promote adaptability to service implementation. Throughout implementation, other facilitation strategies were tailored based on the barriers faced. To ensure comprehensive and transparent reporting of the study, the researchers adhered to three specific reporting guidelines: The “Standards for Reporting Implementation Studies” [43] checklists, the “Recommendations for reporting on ethnographic approaches in implementation research” [44], and the “Recommendations for improved implementation outcomes reporting” [45].

In the present study, the researchers utilized the implementation framework and evaluation models: the “Framework for the Implementation of Services in Pharmacy” (FISpH) [21] and “The Model for the Evaluation of Implementation Programs and Professional Pharmacy Services” by Moullin et al.[25]. This paper evaluates four of six phases of the implementation process: the exploration, preparation, testing, and operation phase (S1 Appendix). The details on the data collection process and the outcome measured for each of the phases are as follows:

*Exploration phase (appraising)* (May 2021-June 2021): Community pharmacists interested in participating in the study were identified through the Malaysian Pharmacists Society (MPS) platform and invited to participate in the study. To be eligible for the study, community pharmacists had to meet specific inclusion criteria: 1) They needed to be registered pharmacists in Malaysia, 2) Working in a retail setting of a community pharmacy in Malaysia, 3) They must agree or have obtained approval from the owner of the pharmacy to pilot the Medication Review service, and 4) They agreed to receive training on Medication Review services in the community pharmacy setting. On the other hand, pharmacists were excluded from the study if they fell into either of the following categories: 1) Provisional registered pharmacists (PRP) or 2) Those who only provided wholesale businesses. They were explained on the study information, including the medication review service, its aims and philosophy, detailed instructions on the workflow process, and their rights in participation. Upon agreement, they were asked to sign the consent form. The outcome measured for this phase was “The number of pharmacies who decided to enroll in the program.” It is important to note that there was no direct or close relationship between the researchers and the study participants.

*Preparation phase (planning)* (July 2021-Sep 2021): During this phase, pharmacists who accepted the invitation attended an online briefing session about the service and study protocol. The protocol included details on the service model and instructions for pharmacists on every step of conducting the service, from recruiting patients to documentation. It also had templates, data collection forms, and patient satisfaction surveys after completing the sessions. The briefing covered the service’s model and steps in conducting the service. Pharmacists were also invited to participate in a workshop on medication review services and communication skills. They were also provided a 2-hour hands-on training experience on conducting medication review services on real patient cases. “The number of pharmacists attending the training” was reported as the outcome of this phase.

*Testing phase (initial operation)* (Oct 2021-April 2022): At this stage, patient recruitment commenced. MMM, a Ph.D. candidate with previous experience in qualitative research, observed the MR services process (provided by the pharmacists) at the settings and took field notes on how the service commenced. Other observations include reviewing the pharmacists’ daily routines and responsibilities, the number of staff on duty and customers visiting the pharmacies, the patient’s journey within the pharmacy, the location of the pharmacy, and the presence of nearby clinics or hospitals. The outcome measured includes “The number of pharmacies that enrolled at least one patient into the program.” Concurrently, pharmacists’ feedback was gathered, and based on their input, adjustments were made to the service model and data collection forms accordingly.

*Operation (implementation)* (Oct 2021-April 2022): Once the service model and data collection form were improved, patient recruitment by the pharmacists at their settings was continued. On-site observation of pre-, during, and post-MR services was done during this study period, and all these aspects were meticulously documented through open-ended field notes. This includes observing how the pharmacists approached and invited patients to avail of the service, their interactions with patients, the review process, and the documentation involved. The onsite visits to each pharmacy study site were done at seven pharmacies, each lasting approximately 3 to 6 hours. Study sites located in different cities (four pharmacies) were not visited due to the movement restrictions imposed during the COVID-19 pandemic. In addition to the onsite observation at the community pharmacy settings, the pharmacists were also given access to open communication platforms such as email and messenger apps. These platforms served as channels for ongoing communication and support. To facilitate continuous feedback and learning, the facilitators conducted weekly online debriefings and discussions with the pharmacists. These sessions allowed them to address challenges faced during the implementation process and collaboratively devise strategies to overcome them. The outcome measured was on "The number of pharmacies that enrolled more than seven patients after six months of commencing patient recruitment."

### The medication review intervention process and evaluation

The priori medication review service model for community pharmacy practice in Malaysia developed by the research team consisted of 3 service levels: Level 1: MR-S (Medication review-standard) aims to develop rapport and engage patients with MR-C and MR-Clin. Level 2: MR-C (Medication review-consultation) is on a walk-in basis and aims to improve patient use and understanding of their treatment and illness. Level 3: MR-Clin (Medication review-clinical) is mostly appointment-based comprehensive and requires all patient data to be available. **P**harmacists in this study recruited adult patients or caregivers using at least one chronic or acute medication. While the model had specific eligibility criteria for patients to qualify for the service, the initial implementation phase did not enforce strict patient selection criteria, such as a predetermined number of medications. This approach was intentional, as one of the goals of the implementation testing was to identify the types of patients enrolled in the study and adapt the medication review process accordingly.

The service workflow commences with pharmacists identifying potential patients using various approaches, such as screening services, assessment of over-the-counter medications, or the dispensing of prescribed medications. Upon recognizing potential candidates, the pharmacist invites the patient to participate, elucidating the service’s objectives based on the specific level of service indicated. Should the patient express willingness to engage in the service, they are then requested to provide consent, which can be in the form of a signed document or a verbal agreement, depending on whether the session is conducted face-to-face or through telepharmacy. The incorporation of tele-pharmacy sessions was also evaluated as part of the model, proving particularly valuable amid the challenges posed by the COVID-19 pandemic. During the sessions, several activities were carried out. These encompassed collecting patients’ demographic information, medical history, and medication history, evaluating their understanding of their condition and prescribed medications, and assessing their adherence to the medication regimen. Subsequently, the process involved identifying any drug-related problems, establishing goals, devising a management plan, and evaluating the necessity for follow-up. The specific activities undertaken were contingent upon the level of service provided, which was tailored to the individual patient’s case and circumstances. For a visual representation of the activities conducted within the medication review service, please refer to the flowchart provided in S2 Appendix. This outlines the steps involved in the process, illustrating how each service aspect is interlinked to ensure effective and patient-centered medication management. Upon the session’s conclusion, patients receive a satisfaction survey for their feedback. The pharmacist subsequently documents the session’s details within the designated database (Refer to S3 Appendix). The evaluation of the effectiveness of the medication review intervention includes the type of services provided (type of MR, time, method of delivery), type of patients attending the service (Demography and characteristics), characteristics of pharmacists conducting the service, and type and number of drug-related problems and interventions provided using the PCNE classification V5.01 [46]. Descriptive statistic was used to analyze the effectiveness of the medication review service outcomes. Frequency, percentage, mean, and SD were used accordingly using SPSS v27.

### Data collection tools

#### Respondents’ data collection form

The pharmacists who provided the services were responsible for filling in essential data for each MR service conducted, including information about the recruitment method used, the type of medication review conducted, and the mode of service delivery—online or face-to-face. An online Google Form, thoughtfully prepared by the researcher, was employed to facilitate this data collection process. An open-text section was also incorporated within the Google Forms, affording pharmacists the opportunity to include any pertinent comments or observations stemming from their interactions with patients or during the service provision. Subsequent to the completion of the MR service, the pharmacists diligently submitted the completed Google Form alongside the patient’s medication review data collection form.

#### Patient data collection form

Data collection forms underwent iterative development and refinement based on pharmacist input, ensuring their ease of use and practicality during the service. The research team trained pharmacists for each updated version, ensuring proficiency in form completion. These adaptations were aimed at aligning the form’s utility with the demanding schedules of community pharmacists, acknowledging their busy workloads. The structure of the data collection form was thoughtfully tailored and adapted from diverse sources, incorporating elements from the home medication review template employed in Malaysia, as well as the medicines use review framework utilized in the United Kingdom. The form encompassed three distinct sections, each serving a specific purpose. The initial section encompassed essential patient demographic details, historical medical and medication information, and an inventory of current medications being taken. The subsequent section delved into drug-related problems (DRPs), encompassing their identification, assigning priority to each issue, and proposing requisite actions for resolution. Additionally, this segment incorporated a consent section that necessitated the patient’s signature, thereby affirming their participation and understanding. The third part is the personal medication record which is made for the patient to keep and can be shared with healthcare professionals and includes a comprehensive list of all medications, including prescription and nonprescription products, herbals, and other dietary supplements. Patients were afforded the choice to receive this record either as a digital copy or in print format. Supplementary appendices in the data collection form included a referral letter template, facilitating effective communication with other healthcare entities, and a PCNE (Pharmaceutical Care Network Europe) classification V5 list for DRPs, serving as a valuable reference tool for pharmacists engaged in the service were also appended to the patient data collection form.

#### Patient satisfaction survey

To gauge patient satisfaction, a survey was devised, incorporating a five-point Likert scale ranging from "strongly agree" to "strongly disagree." This survey was developed drawing from insights from prior studies [41, 47, 48]. The survey aimed to capture patients’ perspectives on various aspects, including their perception of medication and disease knowledge post-session, their appraisal of the pharmacist delivering the service, and their overall contentment with the provided service. Pharmacists were entrusted with administering the survey to patients, after the session’s conclusion, accommodating the patient’s convenience. They could opt to share the survey’s Google form link with patients or provide a physical copy, along with an envelope, which could be returned to the pharmacy’s assistant staff. It is pivotal to underscore that patients’ confidentiality and privacy were strictly safeguarded, ensuring that their candid perceptions remained undisclosed.

### Implementation evaluation (outcome measures and data analysis)

The evaluation of the implementation outcomes encompassed a comprehensive four-phase framework: exploration, preparation, testing, and operation (implementation). These phases were guided by definitions and patient progression criteria established by Varas-Doval et al. for a non-remunerated service, as detailed in Table 1 [38]. In addition to scrutinizing the implementation phases, the study delved into measuring key process outcomes, specifically focusing on dimensions such as reach, acceptability, and fidelity. Comprehensive definitions for these process outcomes, along with the methodologies adopted for their measurement, are also outlined in Table 1. In terms of analyzing preliminary insights into implementation outcomes, a thematic analysis approach was employed. This involved the systematic examination of various data sources, including field notes, interview transcripts, and free-text comments extracted from the survey/data collection forms. Through the coding process, recurring themes or categories began to emerge, characterized by frequently repeated phrases, events, and ideas within the text. Transcripts, email correspondence, and field notes were meticulously examined, with consistent patterns and recurrent subjects being highlighted for further analysis. The insights derived from this qualitative data collection were further subjected to thorough analysis. Researchers MMM, EMH, and MMB engaged in discussions and evaluations, a process designed to ensure reflexivity and enhance the overall credibility of the findings. Barriers and strategies were effectively presented in the stages of a customer-centered service blueprint [49], a representation that aptly illustrated the sequential flow of the service and highlighted key junctures within the customer journey that encountered challenges and were subsequently targeted for intervention (S4 Appendix) [50]. These include physical evidence and patient/customer actions, frontstage actions, backstage actions, and support processes. Additionally, the barriers and strategies documented were categorized and organized following the classification presented by Moussa et al.[31].

**Table 1:** Implementation outcomes evaluated.

Outcome	Definition	Measurement
**Exploration phase**	The number of pharmacies that decided to enroll in the program	Exploration = no. of pharmacies that accepted invitation initially
**Preparation phase**	The number of pharmacists attending the training	Preparation = no. of pharmacists trained and briefed to start the service
**Testing phase**	The number of pharmacies that enrolled at least 1 patient into the program	Testing = no. of pharmacies enrolling 1 patient
**Operation (implementation) phase**	The number of pharmacies that enrolled >7 patients	Operation = no. of pharmacies enrolling >7 patients at 6 months
**Reach**	The absolute number, proportion, and representativeness of individuals who are willing to participate in a given initiative	Reach = total no. of patients enrolled/ target no. patients (7)* 100
**Fidelity**	The degree to which the service is implemented and provided as it was described. **Context fidelity**: Availability of infrastructure and processes necessary to support the implementation of MR	Adherence to the needed infrastructure of pharmacy = Reported by pharmacist and research team (observation)
	**Content fidelity**: Extent to which the pharmacist and team adhere to the intervention components Adherence to intervention Dose: duration and frequency of intervention	Adherence to the MR protocol = Research team evaluation on submission of documents/ patient files/ patient satisfaction survey. Duration of session = reported by pharmacist.
	**Competence fidelity**: Extent to which the pharmacist and team demonstrate skilful delivery of the program Quality of delivery Patient satisfaction: the extent to which a patient engages in or accepts service.	Fidelity of receipt = pharmacist evaluation after each session (good/moderate) Quality of delivery = pharmacist identification of DRP’s identified and interventions made [51] Patient satisfaction survey [48]
**Acceptability**	Service acceptability by patients	Patient satisfaction questionnaire

## Results

### Implementation outcomes

A total of 11 pharmacies across three states in Malaysia participated in the study. Among these, seven were categorized as chain pharmacies, while the remaining four operated independently. Throughout the study period, a total of 54 medication review sessions were successfully conducted, with chain pharmacies contributing 28 sessions and independent pharmacies accounting for 26 sessions. The primary methods employed for patient recruitment include patient-initiated requests for the service (n = 18), proactive invitations extended by pharmacists during medication dispensing or other service interactions (n = 18), and indirect approaches involving patients seeking information related to their medical condition or medication (n = 18). Patient profiles varied, with a higher count of regular patients (n = 32) compared to new patients (n = 21). A majority of the sessions (n = 43) were facilitated on a walk-in basis, while a smaller subset (n = 11) was conducted based on pre-established appointments. Notably, a subset of seven out of the 54 sessions were effectively conducted via tele-pharmacy, an innovative approach that proved particularly valuable during the COVID-19 pandemic. Level 1 and level 2 medication reviews emerged as the predominant types, each accounting for 23 sessions. In addition, level 3 medication reviews were conducted in eight instances, as detailed in Table 2.

**Table 2:** Summary of service characteristics.

Variable	n (%)
Pharmacy type	
Chain	7 (63.6)
Independent	4 (36.3)
**No. of sessions based on the type of Pharmacy**	
Sessions conducted in chain pharmacy	28 (51.9)
Sessions conducted in independent pharmacy	26 (48.1)
**Method of recruitment**	
Customer request for the service	18 (33.3)
Customer request for information related to disease or medication	18 (33.3)
Pharmacist initiated	18 (33.3)
**Patient type**	
New	22 (40.7)
Regular	32 (59.3)
**Service type**	
Walk-in	43 (79.6)
Appointment	11 (20.3)
**Method of delivery**	
Face to face	47 (87)
Tele-pharmacy	7 (13)
**Type of medication review**	
MR-S (level 1)	23 (42.6)
MR-C (level 2)	23 (42.6)
MR- Clin (level 3)	8 (14.8)
**Time conducting the service (mean)**	17.5 (18.42) min: 5 min max: 90 min
**Follow up-appointment arranged**	
Yes	22 (40.7)
No	32 (59.3)
**Pharmacist perception on session**	n (%)
Good	43 (79.6)
Moderate	11 (20.4)

During the initial exploration phase spanning over a span of 2 months, a total of 17 pharmacies displayed keen interest and willingly accepted the invitation to participate in the study. Subsequently, in the preparation phase, 14 pharmacists, translating to 82.35%, actively engaged by attending the training sessions and completing the comprehensive briefing that spanned over 3 months. At the culmination of a six-month period from the initiation of the study’s implementation phase, four (36.3%) pharmacies recruited more than seven patients. The implementation reach was based on two key factors: firstly, the count of pharmacies that embarked on the process of recruiting and actively implementing the service (n = 11); and secondly, the established threshold of minimum patients derived from the insightful study conducted by Varas-Doval, which stands at seven patients per pharmacy for a non-remunerated service. [38] Guided by these considerations, the targeted number of patients to be enrolled in the study was set at 77 (7 patients per pharmacy × 11 participating pharmacies). Applying this calculated benchmark, the implementation reach emerged as 70.12%. This figure was derived by dividing the successfully enrolled patient count (54) by the projected target of 77, subsequently multiplied by 100.

When examining context fidelity within the framework, it becomes evident that the sessions carried a certain degree of flexibility in terms of their physical location. According to the model, these sessions could either take place in a private room or at the pharmacy counter. However, upon observation of the visited pharmacies, it was observed that a majority of pharmacists opted to conduct the sessions at the counseling desk, situated within an open space in the pharmacy environment. Content fidelity was conducted to gauge the alignment between the study’s various stages and the established protocol. In the final stage, patients were provided with satisfaction forms to complete, resulting in the receipt of a total of 54 patient data collection forms. Within this set, a total of 21 patient responses were received, signifying that 38.8% of cases were fully executed in accordance with the stipulated protocol. The sessions were conducted with a mean duration of 17.5 minutes, and a noteworthy 40.7% of patients had follow-up appointments arranged, thereby reinforcing the commitment to comprehensive patient care (refer to Table 2). Feedback from pharmacists regarding the overall quality of session delivery was used to evaluate pharmacists’ competence fidelity. A substantial majority, accounting for nearly 80% (n = 43) of the sessions, was favorably rated as "Good," while the remaining sessions received a "moderate" rating. Pharmacists also demonstrated their proficiency by identifying drug-related problems and implementing appropriate interventions. Moving on to implementation acceptance, which pertains to patient satisfaction, over 80% of patients expressed strong agreement that the medication review session significantly enhanced their understanding of medications and their proper usage. A similar percentage of patients acknowledged the pharmacists’ valuable advice and conveyed their comfort with the interaction. Impressively, more than 90% of patients conveyed their utmost satisfaction with the service, expressing their intent to return and recommending it to friends and family. In terms of patients’ perceptions regarding charges for the medication review service, it is notable that a portion of the patients (12 out of 21) remained unsure about the need for charges. In contrast, six patients expressed agreement with the notion of charges, while three patients did not perceive a necessity for charges (Table 3).

**Table 3:** Summary of patient’s satisfaction on the service conducted (n = 21).

I perceived the service provided has:	Strongly disagree	Disagree	Neutral	Agree	Strongly agree
**Increased my knowledge on my medications and how to take them.**	0	0	0	4(19.0)	17 (80.9)
**Increased my understanding on my health problem.**	0	0	0	5(23.8)	16 (76.2)
**Helped me to understand the importance of taking my medication.**	0	0	0	3(14.5)	18 (85.7)
I am satisfied with the service provided to me.	0	0	0	1(4.7)	20 (95.2)
**Following the service, I found that the pharmacist had:**					
Provided useful advice.	0	0	0	4(19.0)	18 (85.7)
Explained in a way that I can understand.	0	0	0	3(14.5)	19 (90.5)
Was committed to improving my health.	0	0	0	5(23.8)	16 (80.9)
I felt comfortable in my interactions with the pharmacist	0	0	0	4(19.0)	18 (85.7)
**With the service provided, I would:**					
Come back again for the service.	0	0	0	1(4.7)	20 (95.2)
Recommend this service to my relatives and friends.	0	0	0	2(9.5)	19 (90.4)
**Others**			**Yes**	**No**	**Not sure**
I feel that the service should be accordingly charged.			6	3	12

### Implementation barriers and strategies

In the ethnographic and qualitative data collection process, using thematic analysis, barriers were assessed at the meso level (group of pharmacies), and modifiable barriers were targeted with multifaceted strategies in an iterative manner. This involved a series of deliberate actions to overcome challenges and foster successful implementation. At the micro level, which focused on individual pharmacies, pharmacists, and patients, a practice change facilitation approach was employed. This approach was underpinned by a set of strategic interventions and the involvement of practice change facilitators who were well-versed in guiding and supporting transformative changes. The barriers and strategies were presented according to the key components of the service blueprint (S4 Appendix). Similarly, using thematic analysis, the identified barriers and corresponding strategies presented earlier were mapped with the facilitation strategy categories proposed by Moussa et al. [27] (S4 Appendix).

*Physical evidence (Tangible features of each step in the service process) & Patient/Customer actions (Actions performed by a patient when interacting with a service provider)*: When pharmacists began approaching patients to invite them to the medication review service, it became apparent that many patients were unaware of such services. To increase awareness and engagement among customers, a range of strategies were implemented: flyers promotion, personal medication records, pill boxes, optimal session environment with reduced noise and disruption and the most privacy for the patients such as counseling room, tele-pharmacy sessions and targeted patient approaching, for example, those who come for screening services or requesting other professional services such as weight loss or smoking cessation.

*Frontstage actions (Actions that are directly visible to the customer)*: Initially, pharmacists faced challenges integrating the medication review service into their existing routine tasks due to the need for concentrated effort, preparation, and time allocation. The service’s evaluation process added to their workload, involving separate documentation and patient satisfaction forms. This additional administrative burden proved overwhelming and fatiguing for pharmacists, resulting in incomplete satisfaction form submissions. Acknowledging the potential work pressure, facilitators advised pharmacists to prioritize eligible patients for the service, delegate administrative tasks to assistant pharmacists or other staff members, and schedule medication review appointments during less busy periods. These strategies aimed to facilitate smoother implementation and alleviate the challenges posed by integrating the medication review service into pharmacists’ existing responsibilities.

*Backstage actions (Actions done for the customer that are not visible)*: During the initial phase, a platform served as a means of regular communication and support, enabling pharmacists to share difficulties, seek guidance, and receive motivational tips for effective time management, form completion, and customer recruitment were prepared to provide assistance and address any concerns. Pharmacists encountered challenges when it came to completing the necessary forms for the medication review service. To address this issue, Pharmacists with prior experience in delivering medication reviews and those who exhibited a proactive approach to learning were more successful in recruiting patients.

*Support processes (Actions that support frontstage and backstage actions in service delivery)*: Throughout the study, significant efforts were invested in enhancing the patient data collection form to ensure its usability and effectiveness. Feedback from pharmacists played a crucial role in refining the form to make it more user-friendly. The evolution of the data collection form went through several stages: initial offline Word document, online Google Form, and Google Sheets. The latter version aimed to be more user-friendly and convenient for pharmacists. It allowed easy data entry, storage, retrieval, and printing. Each workbook was named after the patient, and it comprised multiple sheets for different aspects of the patient’s information, including patient details, current illness, medication history, personal medication record, and referral letter template. Additional sheets could also be added as needed for follow-up visits.

The main barriers mapped to the barriers and facilitation strategy categories by Moussa et al. [27] are as follows: An inability to plan for change, lack of individual alignment with the change, lack of internal supporters of the change, lack of knowledge and experience related to the change, and lack of monitoring and feedback regarding the change. The main strategies mapped were: Engage stakeholders by creating ownership of the change, equip stakeholders with training, ensure stakeholders contribute to the change, and ensure continuous monitoring of implementation measures. (S4 Appendix)

### Service’s effectiveness outcomes

During the six-month period following the initial implementation phase, a total of 54 patients were successfully recruited for the medication review service. Among these patients, 31 were female and 23 were male. The average age and standard deviation (SD) of the patients was approximately 54.48 (16.36) years. The racial distribution showed a dominance of Chinese patients 30 (55.6%). On average, each patient had 2.26 (1.27) health problems (Table 4). Eleven pharmacists participated in the study, with 7 being female and 4 being male. These pharmacists had varying levels of experience as community pharmacists, ranging from 5 to 23 years. Four out of the 11 pharmacists had prior experience conducting structured medication reviews. Throughout the study, the number of sessions conducted by individual pharmacists varied, with the lowest being two sessions and the highest being ten sessions (Table 5).

**Table 4:** Summary of patients’ demographics and characteristics.

Variable	Mean (SD)
No. of patients	54 patients
Age	54.48 (16.36) min:18 max: 83
Gender	n (%)
Female	31 (57.4)
Male	23 (42.6)
Race	n (%)
Chinese	30 (55.6)
Malay	18 (33.3)
Indian	3 (5.6)
Others	3 (5.6)
No. of health problems	2.26 (1.27)
No. of prescribed medications	4.04 (2.64) min:1 max:11
No. of non-prescribed medications	0.52 (0.83)
Issues raised by the patient	n (%)
Medication-related issue	26 (48.1)
Illness related issue	15 (27.8)
Medication and illness related issue	13 (24.1)

**Table 5:** Characteristics of the pharmacist conducting the medication review services.

No	Gender	Type of pharmacy	Pharmacy location	Staff type	Years of experience	Previous experience in structured MR	Number of MR sessions conducted
1	Female	Big chain	Mall	Pharmacist-in-charge	23	Yes	8
2	Female	Big chain	Local shopping strip	Pharmacist-in-charge	15	No	6
3	Female	Big chain	Mall	Pharmacist-in-charge	12	No	4
4	Female	Big chain	Local shopping strip	Pharmacist-in-charge	20	Yes	4
5	Male	Big chain	Local shopping strip	Pharmacist-in-charge	8	No	4
6	Male	Big chain	Mall	Pharmacist-in-charge	11	No	2
7	Male	Big chain	Mall	Pharmacist-in-charge	5	No	2
8	Female	Independent	Local shopping strip	Pharmacist-in-charge/manager	16	Yes	10
9	Female	Independent	Near hospital	Pharmacist-in-charge	7	No	3
10	Female	Independent	Local shopping strip	Pharmacist-in-charge/manager	10	No	10
11	Male	Independent	Local shopping strip	Pharmacist-in-charge/manager	21	Yes	3

A total of 133 drug-related problems (DRPs) were identified during the 54 medication review sessions that were conducted. The primary type of DRP fell under the "other" domain, accounting for 51.8% (n = 69) of the total DRPs. Within this category, the main issues included insufficient awareness of health and disease (n = 33, 24.8%), unclear complaints (n = 16, 12%), and therapy failure (n = 12, 9%). Other significant DRP domains that were identified included "drug choice problem" (n = 33, 24.8%), where inappropriate drug selection or duplication of therapy occurred. The "adverse reaction" domain accounted for 11.3% (n = 15) of the DRPs, and the "drug use problem" domain represented 9.7% (n = 13). Pharmacists made a total of 64 interventions to address these identified DRPs. The majority of interventions were at the patient level (n = 33, 51.5%), primarily through patient education and counseling (n = 31, 48.4%). To enhance medication adherence for patients with polypharmacy, one intervention involved preparing the patient’s medications in pillboxes. Additionally, since patients often had multiple prescribers and lacked coordination between private clinics and government hospitals, pharmacists provided personal medication records for patients to maintain and share with healthcare practitioners. Interventions were also conducted at the prescriber level (n = 14, 21%), involving referrals to prescribers or other general practitioners (n = 8, 12.5%). At the drug level, interventions accounted for 17% (n = 11) of the total, which included drug/dosage/formulation changes n = 6, 9.3%) and changes in usage instructions (n = 5, 7.8%) (refer to Table 6).

**Table 6:** Number and type of DRPs identified and type of intervention provided according to the PCNE Classification V5.01.

**DRP domain**	**N (%)**	**PCNE Classification**	**N (%) 133 (2.46)**
Adverse reactions	15 (11.3)	Side effects suffered (non-allergic)	15 (11.3)
Drug choice problem	33 (24.8)	Inappropriate drug (not most appropriate for indication)	16 (12)
		Inappropriate drug form (not most appropriate for indication)	3 (2.3)
		Inappropriate duplication of a therapeutic group or active ingredient	3 (2.3)
		Contra-indication for drug (incl. Pregnancy/ breastfeeding)	3 (2.3)
		No drug was prescribed but a clear indication	5 (3.8)
Drug use problem	13(9.7)	Drugs not taken/administered at all	9 (6.7)
		Wrong drug taken/administered	4 (3)
Interactions	3(2.3)	Potential interaction	3 (2.3)
Others	69(51.8)	Patient dissatisfied with therapy despite taking drug(s) correctly	8 (6)
		Insufficient awareness of health and diseases (possibly leading to future problems)	33(24.8)
		Unclear complaints. Further clarification necessary	16 (12)
		Therapy failure (reason unknown)	12 (9)
**Primary Domain**	**N (%)**	**Intervention**	**N (%)** (N = 64)
At prescriber level	14(21.87)	Informed prescriber through telephone, referral letter, sent patients’ medication list	3 (4.6)
		Ask prescriber for information	3 (4.6)
		Intervention proposed immediately through phone	0
		Refer patient to prescriber	8 (12.5)
At patient/carer level	33 (51.5)	Patient education/counselling	31(48.4)
		Written information, medication list provided	0
		Spoken to family member/caregiver	2 (3.12)
At drug level	11 (17.18)	Drug/dosage/formulation change	6 (9.37)
		Instruction for use change	5 (7.8)
		Drug stopped/removed medicines	0
		New drug started	0
Other intervention/solution	6 (9.3)	Side effect report to authorities	2(3.12)
		Refer patient e.g. to physiotherapist, dietician,	1 (1.5)
		Other intervention e.g. compliance packaging etc	3 (4.6)

## Discussion

The current study utilized design thinking methods and implementation Science (IS) principles, which represent a comprehensive approach for guiding the successful implementation of medication review services in community pharmacy settings in Malaysia. IS, being primarily concerned with closing the gap between research findings and their practical application, offered systematic frameworks and models to ensure that medication review services as evidence-based practices are effectively integrated into real-world healthcare settings [21, 52]. Design thinking, on the other hand, is a human-centered problem-solving approach. It places a strong emphasis on understanding the needs and preferences of end-users such as pharmacists and customers. This empathetic approach can lead to the development of services that are more likely to be accepted and used [53, 54]. This synergy between the two approaches ensures that implementation strategies are not only evidence-based but also tailored to the unique needs and preferences of the end-users [55].

This study represents a study of an effectiveness-implementation hybrid type 3, focusing on the implementation of a medication review service intervention within the community pharmacy setting in Malaysia. Leveraging an ethnographic methodology, the study aimed to comprehensively outline, assess, and refine a customized medication review service blueprint that has been implemented and simultaneously evaluated the efficacy of the implementation strategies employed. This approach seeks to enhance the understanding and integration of evidence-based practices in real-world settings, bridging the gap between theory and practice. Moreover, in accordance with the recommendations of implementation science, the strength of this study lies in the coherent utilization of established terminologies across both the framework and the outcomes of implementation [45, 48, 56, 57].

Over the course of the 12-month implementation study encompassing the exploration, preparation, testing, and operation phases, the initial two phases (exploration and preparation) extended for a period of five months before the commencement of patient recruitment. This timeline underscores the significance of the efforts and strategies deployed during these initial stages, showcasing their substantial influence on the subsequent phases that encompassed the official initiation of patient recruitment and the actual implementation of the medication review intervention. Similar findings were highlighted in Garcia’s study, underscoring the pivotal role of implementation frameworks in guiding successful implementation [47]. The majority of patients’ cases were categorized as level 1 MR or level 2 MR, primarily conducted on a walk-in basis. This implementation approach appears most viable within the context of the community pharmacy landscape in Malaysia, where the practice of referral is not commonplace. It is anticipated that the prevalence of level 3 MR (appointment-based) cases will likely increase with extended implementation duration and patient follow-up.

After six months of patient recruitment, the implementation reach stood at 70%, a finding that aligns with a similar study conducted by Varas Doval, where the reach was reported as 75% after seven months. Notably, within the context of this study, 78.5% of pharmacies progressed to the testing phase, while 36% successfully transitioned to the implementation phase within the same six-month timeframe. In contrast, Varas-Doval’s study showed significant results, with pharmacies achieving full implementation only after a year of initiating the implementation process [38, 47]. These findings harmonize with Blanchard et al.’s insights, suggesting that a typical service takes around 2–4 years to achieve full implementation [22]. In the context of comprehensive medication management (CMM), as described by Blanchard et al., full implementation is characterized by at least 50% of eligible patients receiving CMM services with fidelity and achieving the intended outcomes. As such, the attainment of full implementation and sustained integration is anticipated to evolve over an extended study duration, marked by ongoing dedicated efforts.

Implementation fidelity was assessed using various measures, revealing distinct outcomes for content fidelity (38.8%) and competence fidelity (80%). However, it is important to note that the assessment of content fidelity might have underestimated the actual level due to constraints associated with the final stage of patient feedback collection. This could be attributed to time limitations and challenges in completing surveys immediately post-service. Pharmacists’ evaluation of their service delivery exhibited a range of scores, spanning from moderate to good, a variation whose underlying reasons remain unclear. It is plausible that prior experience in implementing structured medication reviews could have influenced these results, although the precise factors driving this variation warrant further investigation. Conversely, the competence fidelity aspect demonstrated encouraging results, with pharmacists successfully identifying 133 drug-related problems (DRPs) and implementing 64 interventions across 54 patients. The average duration of the medication review sessions, at 17.5 minutes, was deemed reasonable and in line with similar studies, particularly since the majority of cases pertained to less comprehensive MR levels 1 and 2. While the response rate to the post-service satisfaction survey was relatively modest (21 out of 54, 39%), the outcomes conveyed a high level of satisfaction and interest among patients toward the provided service. Nevertheless, the study unearthed a certain uncertainty among patients regarding their willingness to pay for these services out of pocket. A prior survey in Malaysia indicated that over half of the patients expressed a willingness to pay for pharmacy professional services; however, this survey focused on dispensing services, which are less intricate than medication review services [58].

One crucial facet of implementation studies involves aligning strategies with the goal of overcoming identified barriers to service delivery. A prior study, which forms a part of the larger Medication Review Project for Malaysia, examined the macro-level barriers and strategies, while the current paper delves into the meso and micro barriers encountered during the implementation testing phase [12]. Noteworthy barriers observed across the various components of the service encompassed pharmacists’ challenges in planning for change, a lack of alignment with the proposed changes, and an absence of robust monitoring and feedback mechanisms. While pharmacists exhibited keen interest and enthusiasm for receiving training and implementing the service, the practical task of commencing patient recruitment and prioritizing the service alongside their existing demanding daily duties posed a significant challenge. This initial overwhelm led to a dropout rate of 6 out of 17 pharmacists, a trend in line with similar findings from studies conducted in the UK, Spain, Belgium, and Australia [59]. Lelubre et al. contextualized these challenges through the lens of Rogers’ theory, which underscores the need for continuous support, training, and feedback to bolster the self-efficacy of "laggard" pharmacists [37, 60]. The facilitation strategies adopted in response aimed to empower and engage pharmacists, enabling them to navigate the workflow seamlessly, enhance their confidence in service implementation, and cultivate public awareness. These strategies were implemented through continuous support mechanisms, training workshops, demonstration sessions, and on-site assistance. A pivotal component of these facilitation strategies was the medication review data collection form, which was instrumental in guiding the service’s flow and execution. This form underwent multiple revisions to simplify and streamline its use for pharmacists in conducting and documenting the service. This mirrors a web-based data collection form strategy employed for Medication Therapy Management (MTM) services in the USA, which highlighted that the introduction of a novel pharmacy information system necessitates robust implementation support to assist end-users in familiarizing themselves with program features, integrating the software into their workflow, and optimizing its usage to enhance patient care [61].

In this study, a deliberate decision was made not to impose specific patient eligibility criteria, aiming to gain insight into the patient population naturally attracted to the service. Notably, elderly patients emerged as potential candidates, evident from the mean age of 54 years among recruited participants. The study’s findings also revealed that the average number of health conditions or diseases per patient was two, accompanied by a mean of four medications. In contrast, established medication management services like MTM in the United States set eligibility criteria requiring patients to be on at least four medications and have chronic conditions [62]. Similarly, the Medicines Use Review (MUR) service in the United Kingdom focuses on patients using specific high-risk medications such as NSAIDs, while the Malaysian MTAC targets patients with designated health conditions like diabetes, chronic kidney disease, and those undergoing warfarin therapy [5, 63]. In the context of Malaysia’s community pharmacies and the diverse range of patients they serve, the current study highlighted that medication reviews may encompass broader eligibility criteria compared to other aforementioned services. The most commonly identified drug-related problems fell within the "others" domain of the Pharmaceutical Care Network Europe (PCNE) classification V5.01, predominantly involving patients’ awareness of their diseases and medications. "Drug choice problems" represented another significant domain. These findings parallel a study conducted in German community pharmacies [64], although they diverge from observations within hospital settings or collaborative medication review models [65]. This divergence is possibly attributed to the nature of medication reviews conducted in this study, which predominantly focused on level 1 and 2 MR cases, lacking extensive access to laboratory data and comprehensive medical histories. The interventions carried out by pharmacists predominantly occurred at the patient level, involving counseling and education. Notably, 21% of the interventions extended to the GP level, where pharmacists either referred patients to GPs or composed referral letters. This development is noteworthy, as it holds promise for enhancing communication between pharmacists and GPs, which is often limited [41]. Finally, the ultimate aspiration is to reach a state of sustainability where the true impact of medication review services on patient outcomes, encompassing clinical, humanistic, and economic dimensions, becomes evident [52]. It is of utmost importance to maintain the continuous delivery of medication review services. Prematurely discontinuing them might entail missing out on their potential benefits, both in terms of public health and clinical healthcare [66, 67]. Therefore, as we strategize for implementation, we must concurrently contemplate how to ensure the intervention’s long-term viability, marking a significant stride forward in this field.

## Limitations

While this implementation study may be considered limited in scale and duration compared to similar efforts in Belgium and Spain [37, 38], it is important to note that the Tennessee Medicaid medication therapy management implementation program recruited a considerably low number of pharmacists for qualitative interviews, despite the MTM program involving multiple organizations and payers [39]. Another aspect to consider is that this study lacks the measurement of patient recruitment outcomes at different time points, potentially limiting a comprehensive understanding of the process. Furthermore, the fidelity assessment conducted may not encompass the full spectrum of evaluation tools, highlighting the possibility for future research to incorporate additional methods for ensuring the accurate implementation of medication review services in the Malaysian community setting. It is worth mentioning that the study was conducted within the context of the COVID-19 pandemic, which could have influenced the results to some extent. Nonetheless, it is commendable that the pharmacists were appropriately supported and guided to implement the service within their respective settings, indicating a strong commitment to overcoming challenges even during uncertain times.

## Future studies

Future studies could involve conducting type 2 hybrid studies with an extended duration and a broader scale. Emphasis could be placed on augmenting the exploration and preparation phases by incorporating additional methods, such as assessing pharmacist readiness and clinical knowledge. This approach would offer a more comprehensive understanding of the implementation process beyond just gauging their interest. It would be beneficial to integrate a range of assessment tools, surveys, and questionnaires to gather comprehensive data [23, 39, 68]. Future studies could also focus on the development of an adapted pharmacy software that seamlessly integrates various tools and resources for the medication review service. This could encompass a web platform and a tool for detecting drug-related problems (DRPs), resulting in a more streamlined and effective service delivery. There is also a potential avenue for involving patients in the research process. This could entail conducting interviews or surveys with potential beneficiaries of medication reviews. By seeking patient perspectives, researchers can gain insights into the actual needs, preferences, and experiences of individuals who could benefit from these services.

## Conclusion

This pilot study (implementation testing) represents a significant milestone in the successful implementation of medication review services in Malaysia. It played a crucial role in shaping and optimizing the medication review model and data collection methods to align with the unique dynamics of community pharmacy settings in the country. By integrating design thinking principles and implementation science frameworks and by tailoring strategies from the early stages of service innovation and blueprint design, a structured pathway for effective implementation and widespread adoption of the service has been established. This approach holds promise for ensuring the success and long-term sustainability of medication review services within the community healthcare landscape of Malaysia. Employing a multi-faceted approach that encompasses various levels and components, supported by strategic partnerships, meticulous planning, ongoing monitoring, feedback mechanisms, and continuous refinement and adaptation, is essential to harnessing the potential of medication review services in Malaysia fully. This comprehensive approach is poised to drive the advancement of medication review practices in the country and contribute to enhanced patient care and health outcomes.

## Supporting information

S1 Appendix Implementation Phases and data collection points. (DOCX)

S2 Appendix Flowchart of the activities conducted in the medication review service. (DOCX)

S3 Appendix Summary of the data collection process and documentation. (DOCX)

S4 Appendix Barriers and strategies during the implementation testing. (DOCX)

S1 Data (XLSX)
