# Supplementary material for: Integrating design thinking and implementation science principles in delivering a medication review service in the community pharmacy setting—An implementation testing study
Source: PLoS One. 2024 Jun 13;19(6):e0304291. doi: 10.1371/journal.pone.0304291 (PMC11175411; doi:10.1371/journal.pone.0304291)
Supplement: S1 Appendix — (DOCX) [file pone.0304291.s001.docx]

**S1 Appendix.** Implementation Phases and data collection points

*Medication review observation and identification of DRPs and interventions


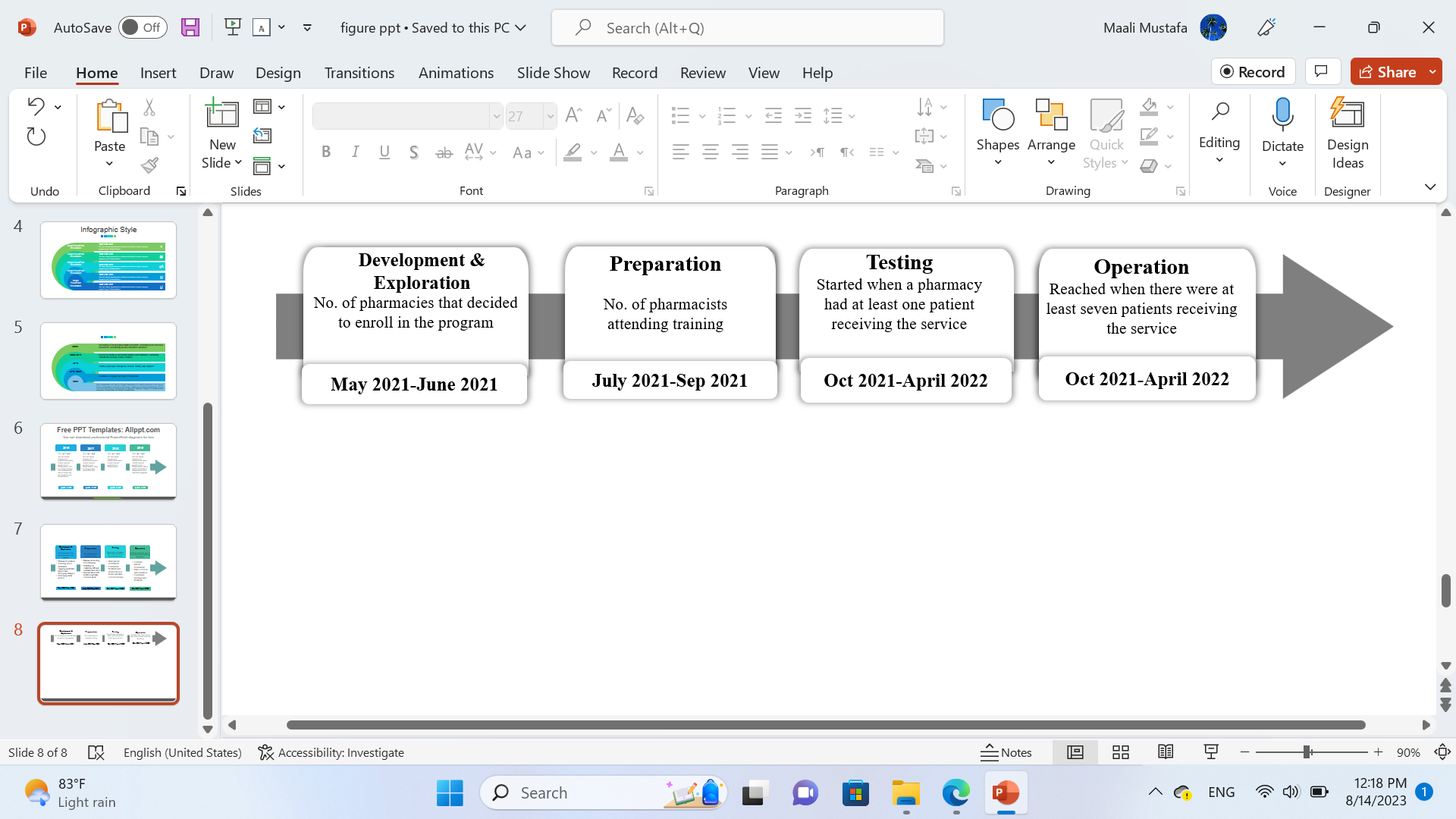


Discovery

Full implementation and sustainability

*Implementation phases, reach, fidelity, acceptance, meso strategies

Stages of the service

**Pre-service Service Post-service**

**Patient actions**

**Frontstage actions**

**Backstage action**

**Support**

*Barriers, micro strategies

***Points of data collection**
