# Supplementary figures and images for: Integrating design thinking and implementation science principles in delivering a medication review service in the community pharmacy setting—An implementation testing study
Source: PLoS One. 2024 Jun 13;19(6):e0304291. doi: 10.1371/journal.pone.0304291 (PMC11175411; doi:10.1371/journal.pone.0304291)

**S2 Appendix.** Flowchart of the activities conducted in the medication review service.


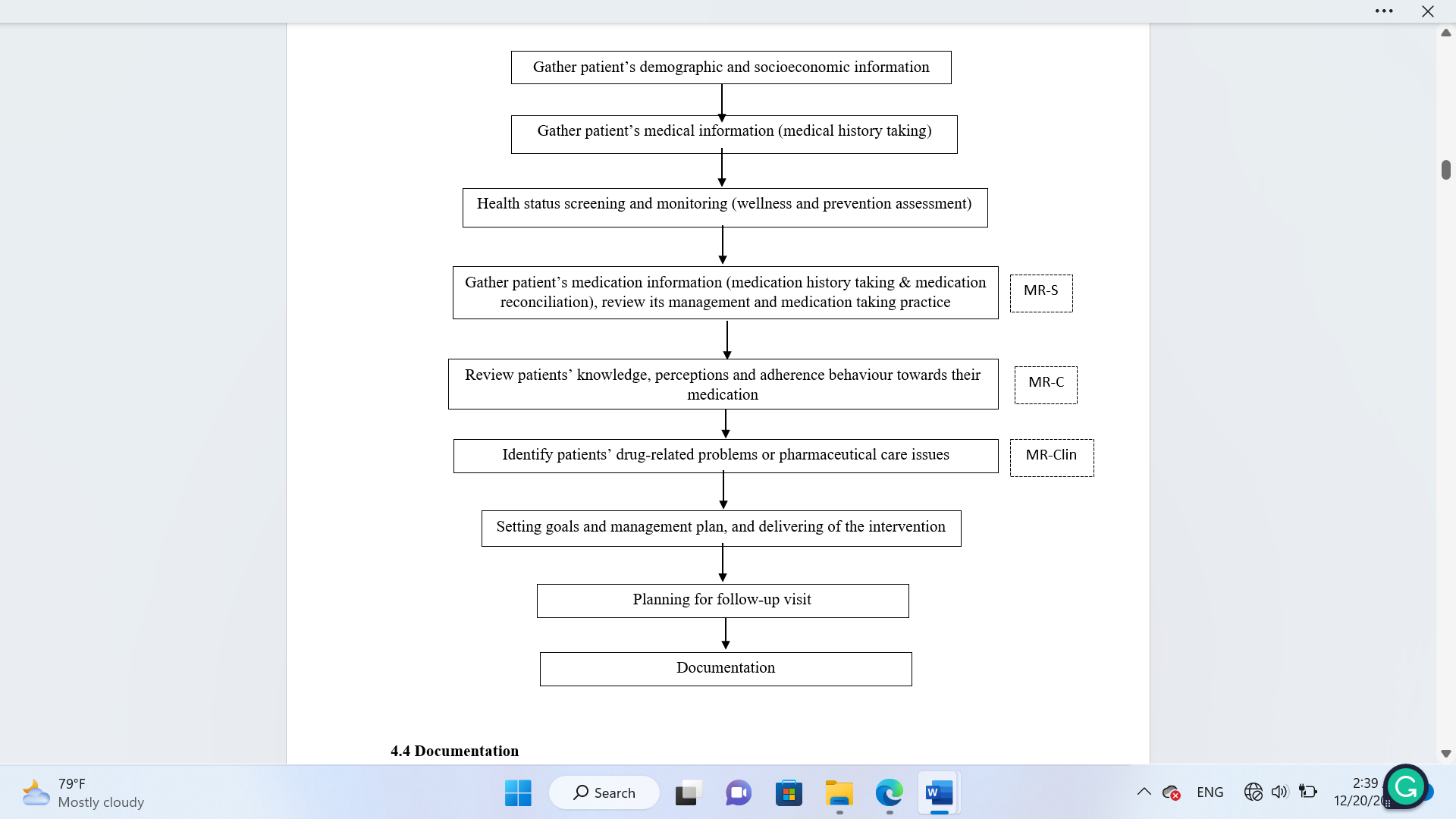

Supplement: S2 Appendix — (DOCX) [file pone.0304291.s002.docx]

**S3 Appendix.** summary of the data collection process and documentation


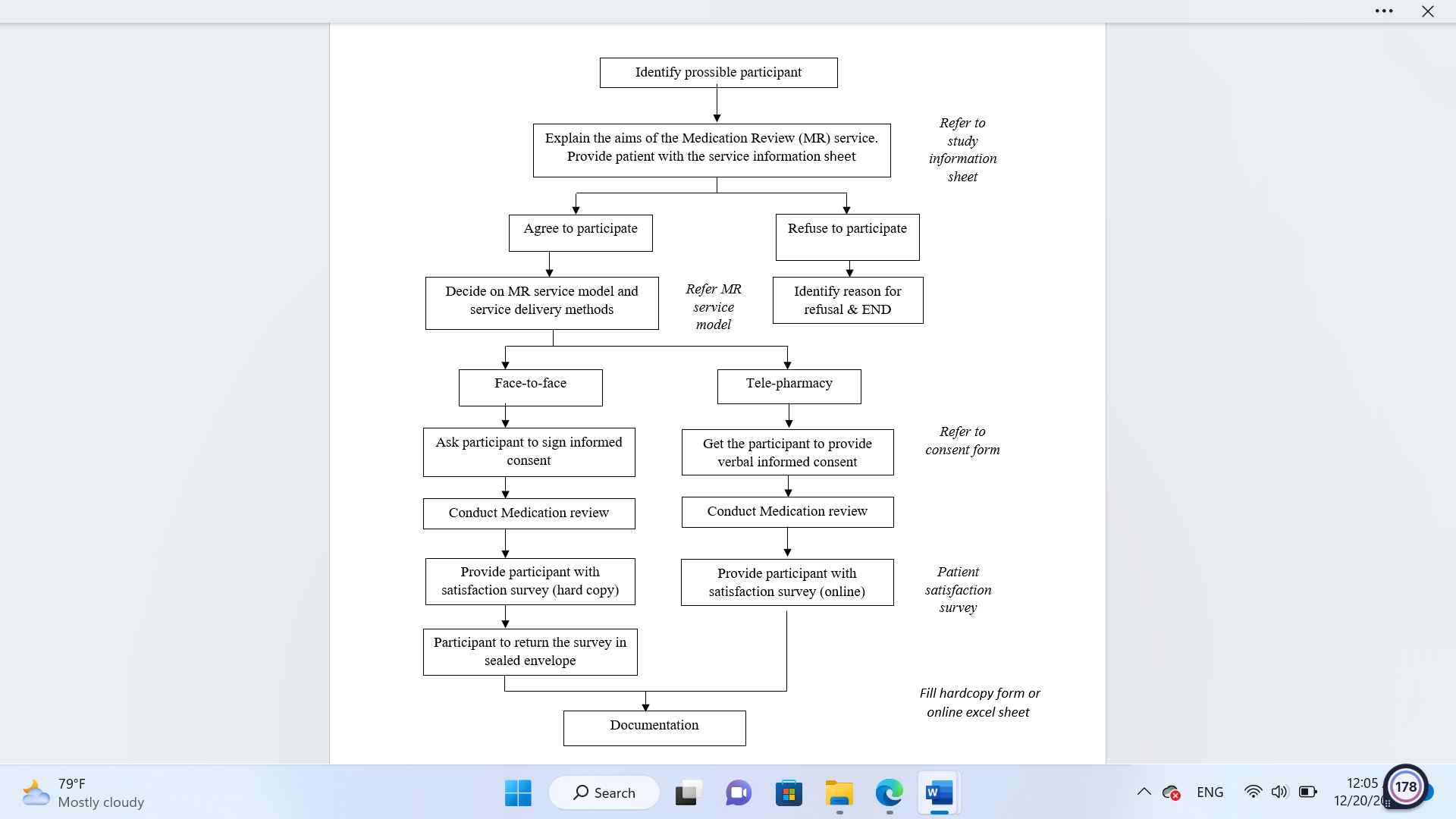

Supplement: S3 Appendix — (DOCX) [file pone.0304291.s003.docx]
