# Supplementary material for: Integrating design thinking and implementation science principles in delivering a medication review service in the community pharmacy setting—An implementation testing study
Source: PLoS One. 2024 Jun 13;19(6):e0304291. doi: 10.1371/journal.pone.0304291 (PMC11175411; doi:10.1371/journal.pone.0304291)
Supplement: S4 Appendix — (DOCX) [file pone.0304291.s004.docx]

**S4 Appendix**. Barriers and strategies during the implementation testing

| Component | Pre-service | Service | Post-service | Mapping of barriers and strategies under the Categories from Moussa et al. |
| --- | --- | --- | --- | --- |
| **Physical evidence**  Tangible features of each step in the service process | B: Patient unaware of service, pharmacist poor communication skills  S: Engaging patients, MR flyer was given to patients  S: Present/service details on the pharmacy banner | B: Patient poor adherence and knowledge on medication/ pharmacist poor skills  B: Multiple prescribers  S: Provide the patient with personal medication Record/Pill box  Educational material | B: Patient poor adherence and knowledge on medication/ pharmacist poor sills  B: multiple prescribers  S: Provide the patient with personal medication chart/Pill box  Educational material | B: An inability to plan for change  **S: Equip stakeholders with training**  Provide/recommend skills/technical training  Provide Knowledge training  Encourage discussion of training topic as a group (workshop) |
| **Patient (Customer actions)**  Actions performed by patient when interacting with a service provider | B: patient unaware of service.  S: Flyer sent to regular patients through WhatsApp | B: unsuitable pharmacy structure for MR session  S: prepare private room or corner in the pharmacy for comfort of patient | B: unknown patient perception of service  S: Follow up and ask for patient feedback/ Patient satisfaction survey | B: An inability to plan for change  A lack of individual alignment with the change  **S: Equip stakeholders with training (as above)** |
| **Frontstage actions**  Actions that are directly visible to the customer | B: Pharmacist busy with many customers/administrative work.  S:  Try to prioritize patients that are potential and eligible for service.  Delegate other patients to other staff.  Schedule appointments in the least busy hours of the day  B: front staff and pharmacists are having difficulties in conducting service/have no time to approach patients and invite them to the service  S: Pharmacist and staff briefing on service  S: WhatsApp group for researchers and pharmacists to communicate and give feedback, motivate pharmacists | B: Poor skills in conducting MR  S: Pharmacist attended a workshop on Medication review  B: pharmacists feeling unprepared to conduct the MR session  S: online demo session was conducted by a pharmacist with MR experience. Other pharmacists in the study attended to learn and get motivated to start recruitment.  S: checklist for pharmacists for tasks to be completed | B: lost follow-up  S: send reminders  S: Call and conduct online sessions | B:An inability to plan for change  A lack of internal supporters of the change  A lack of individual alignment with the change  S: **Engage stakeholders by creating ownership of the change**  Encourage collaboration and teamwork  Recommend or aid in conducting a performance review  **S: Equip stakeholders with training**  Conduct/ recommend role-playing/role modelling  S: **Ensure stakeholders contribute to the change**  Acknowledge ideas  Encourage knowledge/experience sharing  **S: Feedback progress of implementation measure**  Organise or conduct meetings (face-to-face)  Lead virtual meeting (coach present digitally e.g. webinar or skype  Provide constructive feedback  Acknowledge success/recognise/celebrate achievements  Provide ongoing encouragement |
| **Backstage actions**  Actions done for the customer that are not visible | B: difficulty in preparing for the MR session  S: Pharmacist attended workshop on Medication review | B: difficulty in filling data collection forms  S: researcher visiting pharmacy and providing on-site support and training.  S: WhatsApp group for researcher and pharmacists to communicate and give feedback, motivate pharmacists | S: WhatsApp group for researcher and pharmacists to communicate and give feedback, motivate pharmacists | B: A lack of knowledge and experience related to the change  An inability to plan for change  S: **Equip stakeholders with training**  Conduct/ recommend role-playing/role modelling  **B:** A lack of individual alignment with the change  A lack of internal supporters of the change  S: **Create buy-in amongst stakeholders**  Ask about and address specific individual concerns regarding the change  B: A lack of monitoring and feedback of the change  S: **Ensure continuous monitoring of implementation measures**  Monitor service provision  Monitor Staff measures |
| **Support processes**  Actions that support frontstage and backstage actions in service delivery | B: poor data collection methods and IT resources  S: Having a computer to access notes, websites, and document patient MR sessions | B: poor data collection methods and IT resources  Feedback to research team and modification to data collection forms | B: poor data collection methods and IT resources  S: training on conducting online MR sessions and follow up with patient. | B: A lack of knowledge and experience related to the change  A lack of monitoring and feedback regarding the change  S: **Feedback progress of implementation measure (as above)**  B: Undefined change objectives and lack of objective feedback  S: **Communicate the change to stakeholders**  Inform the entire group of the change and objectives verbally  Inform individuals of the change and objectives verbally |

B: Barrier S: Strategy
